# Use of Proteomics in the Study of Mastitis in Ewes

**DOI:** 10.3390/pathogens8030134

**Published:** 2019-08-29

**Authors:** Angeliki I. Katsafadou, Natalia G. C. Vasileiou, George C. Fthenakis

**Affiliations:** Veterinary Faculty, University of Thessaly, 43100 Karditsa, Greece

**Keywords:** cathelicidin, diagnosis, pathogenesis, mastitis, sheep, subclinical mastitis

## Abstract

The objective of this review is to describe the usage and applicability of proteomics technologies in the study of mastitis in ewes. In ewes, proteomics technologies have been employed for furthering knowledge in mastitis caused by various agents (*Staphylococcus aureus*, *Staphylococcus chromogenes*, *Mannheimia haemolytica*, *Streptococcus uberis*, *Mycoplasma agalactiae*). Studies have focused on improving knowledge regarding pathogenesis of the infections and identifying biomarkers for its diagnosis. Findings have revealed that ewes with mastitis mount a defence response, controlled by many proteins and over various mechanisms and pathways, which are interdependent at various points. Many proteins can participate in this process. Moreover, as the result of proteomics studies, cathelicidins and serum amyloid A have been identified as proteins that can be used as biomarkers for improved diagnosis of the disease. In the long term, proteomics will contribute to improvements in the elucidation of the pathogenesis of mastitis. Further in-depth investigations into the various proteomes and application of new methodological strategies in experimental and clinical studies will provide information about mastitis processes, which will be of benefit in controlling the disease. Improvement of diagnostic techniques, establishment of prognostic tools and development of vaccines are key areas for targeted research.

## 1. Introduction

The entirety of all proteins that exist in a cell or a tissue at a particular time is termed ‘proteome’—this term encompasses all post-translational modifications that occur. Proteomes are dynamic entities, and changes in their composition depend upon physiological conditions and pathological situations and processes, which occur in a particular tissue of that organism. Proteomics studies aim to identify proteins present in tissue samples during various physiological states, as well as to quantify changes in protein abundance during the pathological conditions under study. There are several methodologies to achieve the above aims that depend upon the type of sample and the equipment available [1].

Large-scale studies that measure the characteristics of families of cellular molecules (e.g., genes, proteins, metabolites) implementing high-throughput technologies, are characterized collectively by the use of the suffix ‘*-omics*’ at the end of the name of the characteristic measured. This term refers to methodologies and techniques that investigate the various roles of the molecules making up cells within a tissue, their actions, as well as the relationships and interdependencies between them [2]. Collectively, these technologies have been defined as ‘*biomics*’ [3], as they support the investigation of tissues and organisms at varying levels.

In particular, proteomics is defined as the large-scale study of protein expression, protein–protein interactions or post-translational modifications [4,5]. On this basis, the dynamics of the response(s) of cells and tissues to changes in their microenvironment within these tissues can be evaluated and recorded. Moreover, changes in protein presence or abundance, interaction or modification can be studied, as these result from differing normal states or pathological processes within a tissue or organism.

**Figure 1:**
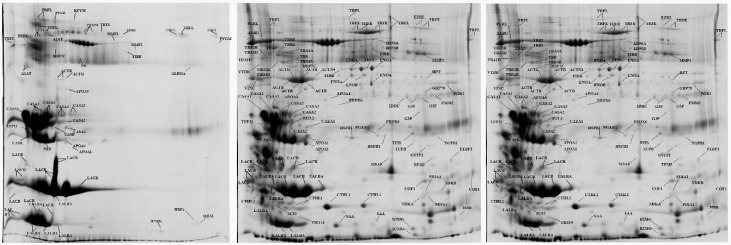
Annotated 2-DE (two-dimensional electrophoresis) gels from milk whey samples collected sequentially from a mammary gland of a ewe; left: sample collected before deposition of *Mannheimia haemolytica* into the ipsilateral teat of the ewe; centre: 12 h after bacterial deposition; right: 1 day after bacterial deposition (protein identification performed by use of a matrix-assisted laser desorption/ionization time-of-flight mass spectrometer (MALDI-TOF MS) [1].

Recently, various ‘*-omics*’ sub-disciplines have been developed. These focus on particular categories, such as epigenomics, lipidomics, interactomics and foodomics [6]. Furthermore, the term ‘*veterinomics*’ has been coined to describe ‘*-omics*’ approaches in all branches and facets of veterinary medicine [7]. These may be employed, among others, to evaluate differences between healthy and diseased animals, and to establish biomarkers for potential diagnostic purposes. During the course of diseases, proteins and/or pathways change [8,9] (Figure 1 [1]) and thus are targets for future research. The findings help to improve knowledge regarding causal bacteria–animal interactions for the identification of proteins, which participate in mastitis processes, such as in the elucidation of interactions, the establishment of biomarkers and the potential development of vaccines [7].

The disease is widespread in sheep flocks. In a recent extensive field study of ovine mastitis across Greece, Vasileiou et al. [10] found its prevalence to be 26%. In other studies in Spain and Turkey, the prevalence of the disease was 34% and 18.5%, respectively [11,12]. Relevant findings in Italy vary widely from 10% to 50% [13], whilst no recent field studies are available from France [13].

The European Food Safety Authority has indicated that mastitis reduces welfare of affected ewes in all types of sheep production (meat, milk, wool) and in all systems of flock management [14]. Mastitis leads to anxiety, restlessness, pain and changes in feeding and behavioural patterns in affected ewes [15], which raises welfare concerns. The disease also causes significant adverse financial effects, which can be summarised as follows: (a) the need to cull affected ewes and to purchase replacement animals; (b) in dairy production flocks, reduced quantity and suboptimal quality (to the point of rejection) of milk from ewes with mastitis and (c) in meat producing flocks, reduced growth rate and suboptimal bodyweight of lambs of affected ewes. According to Giadinis et al. [16], mastitis is the primary cause (over 85% of all incidents) of the ‘milk-drop syndrome’ in ewes, which refers to >30% reduction of flock milk production, with >25% of ewes in the flock affected, each ewe with >25% reduction in milk yield [16].

The objective of this paper is to review the usage and applicability of proteomics technologies in the study of ovine mastitis. Descriptions of proteomics techniques that can be used in such studies have been presented before [1,17,18] and are outside the scope of this paper.

## 2. Use of Proteomics in the Study of Milk Production in Healthy Animals

### 2.1. General Considerations

Milk is a complex biological fluid with micro-quantities of various substances that fulfils roles for the offspring and the mammary gland itself. These refer to nutrients (e.g., vitamins) and to antimicrobial and immunoregulatory agents (e.g., immunoglobulins, cytokines, chemokines) [19]. Sheep milk is an important food because of its high biological value. It is consumed mostly, after appropriate processing, as dairy foods (e.g., cheese, yoghurt). Milk has been the target of extensive proteomics investigations by means of relevant techniques [20].

Each animal species has a unique pattern of proteins in their milk [21,22], thus it is possible that the technology could be used for distinguishing samples of milk according to the animal species that it is sourced from. The respective patterns have been established through conventional or proteomics techniques, but it is noteworthy that improvements in proteomics technology will lead to further discoveries of proteins in milk [6].

Proteomics can be used also for studying the suitability of milk for processing in preparation of dairy products, in the light of improving human nutrition, which has been termed ‘*foodomics*’. In this context, Anagnostopoulos and Tsangaris [23] produced details of the proteome of ‘feta’ cheese, a traditional sheep milk product made in Greece [23].

In proteomics studies of milk, a technical difficulty is that there are some proteins with higher concentration therein (e.g., caseins); this may obscure detection of proteins with low abundance. With technical developments (e.g., the use of new-generation mass spectrometers) and advancements in bioinformatics, it can be expected that products from proteomics investigations will be improved.

Proteomics analyses of milk can be divided into evaluations of whole milk, of caseins (the most abundant protein of milk), of whey and of the protein fraction of the milk fat globule membrane [24]. Modern proteomics and bioinformatics technologies can be employed to fully characterise and map milk proteomes or, otherwise, to provide details regarding differentially expressed proteins in milk. In any case, it is appropriate to mention that successful proteomics investigations depend upon availability of complete databases, which would include gene and protein sequence information for the animal species of interest [6,25].

Other studies refer to applying proteomics methodologies in studying the mammary gland and milk secretion during infection (Section 3). Results of such investigations can contribute to the elucidation of pathogenesis of mastitis, that way improving its control, as well as pointing out possible biomarkers that can advance diagnostic approaches to the disease.

### 2.2. Appraisal of Proteomics Studies in Milk

The study of milk production and milk of ewes has been greatly facilitated by proteomics technologies [21,26]. Different researchers have worked on varying fractions of milk. Relevant works include the study of protein synthesis during lactation [27], the comparison of ewes’ milk with that of cows [28] and the study of proteins in the milk of indigenous sheep breeds [6,29]. Further studies have investigated and elucidated enzymes involved in milk synthesis and the formation of milk lipids [30,31], as well as the metabolism of glucose or lactose during lactation [32]. Other studies have evaluated the milk fat globule membrane [33,34,35,36], the whey fraction of ewes’ milk [6,29,36] or milk samples from ewes with mammary infection (Section 3).

The varying strategies reflect different objectives on the part of the researchers. Whey includes cell-secreted proteins [19]; it also contains proteins relevant to innate immunity and acute-phase response proteins [36]. Milk fat globules originate from milk-producing mammary epithelial cells and are surrounded by their endoplasmic reticulum and apical plasma membranes; cytoplasmic proteins are often trapped between these membrane layers [37,38], determining milk fat globules as a reasonable target in studies of mammary cells [36].

Ewes’ colostrum [39,40] and whey from colostrum [41] have also been studied extensively. Moreover, the protein intake by newborn lambs after colostrum ingestion has been studied by looking into the protein profile in lambs’ blood plasma [42]. This contributes to determining the degree of passive immunoprotection of lambs.

The proteomics approach can be a useful tool for investigating the physiology of the mammary gland during lactation or the dry-period, and for unravelling features unique in the various sheep breeds (which may also reflect nutritional habits across breeds). The latter approach would also support conservational aspects of dairy animal biodiversity. Proteomics can also be used for assessing milk quality, which helps in the design of novel dairy products from ewes’ milk.

Moreover, a proteomic and peptidomic approach in evaluation of milk would contribute in controlling food adulteration [18,43], developing traceability methods and finding nutraceutical properties of milk and milk products [22]. Correct interpretation of proteomics findings would support the dairy industry in the development of functional food proteins from sheep milk [19,22].

## 3. Proteomics Studies in Mastitis in Sheep—Significance of Findings for the Study of the Infection

There are few studies presenting proteomics work in mastitis in ewes. These refer to providing an accurate and early detection of the infection, to establish a correct identification of causative agents and to evaluate animal–pathogen interactions and animal immune responses.

Two-dimensional polyacrylamide gel electrophoresis (2-D PAGE), immunoblotting and spot sequencing has been used by Le Maréchal et al. [44] with the aim of obtaining a description of the immunogenic proteins of *Staphylococcus aureus* in the blood of ewes with mastitis [44]. In this way, it has become possible to establish the core seroproteome of the bacterium (defined as the entirety of proteins present in all *S. aureus* strains), as well as its accessory seroproteome (defined as the bacterial proteins that vary depending upon the *S. aureus* strain and the ewe infected). With this, proteins of the bacterium surface were resolved (by using sodium dodecyl sulphate-PAGE [SDS-PAGE]) and transferred onto a membrane. Then, they were incubated with different blood serum pools from ewes that had mastitis caused by *S. aureus* [44]; by using this methodology, it was possible to identify the most immunoreactive staphylococcal proteins. Finally, the authors found that proteins from *S. aureus* harvested from culture media during their growth phase were more immunogenic than were other proteins detected from the same bacterial isolates during their stationary phase. That way, it was possible to explain the results of Fthenakis and Jones [45], who in previous experimental inoculation studies found that intramammary inoculation of ewes with culture of staphylococcal strains that had been incubated for 5 hours (i.e., obtained during the growth phase of the challenge strain) resulted consistently in mastitis. During serological proteome analysis (SERPA) of ewes that had developed mastitis, it was possible to identify 89 immunogenic proteins in *S. aureus*; of these, 74 proteins were identified to form the bacterial core seroproteome [46]. Specifically for *S. aureus* mastitis, Seyffert et al. [47] indicated N-acetylmuramyl-L alanine amidase as an immunoreactive protein of significance in the host reaction to infection. The results have a potential significance in the development of immunological tools against *S. aureus* mastitis.

With regard to mammary infection during contagious agalactia (*Mycoplasma agalactiae*), Addis et al. [33] studied the proteomic profiles of the milk fat globule membrane in infected ewes. This led to detection of several proteins that were found to be involved in inflammation, chemotaxis of immune cells and antimicrobial defences, including cathelicidins and calprotectin (S100-A8/S100-A9). Furthermore, the findings suggested the participation of mammary epithelial cells in the defensive response of the animals against pathogens. These authors concluded that various pro-inflammatory proteins (e.g., S100 proteins, cathelicidins) were secreted by the mammary epithelial cells and played a role in the defence of the mammary gland against invading bacteria [33]. The same authors also studied cathelicidins and calprotectin subunit S100-A9 within mammary tissues and indicated the ability of epithelial cells of the mammary gland to secrete and release various molecules participating in the defence response of the host [33]. It is noteworthy that the hypothesis of the mammary epithelial cell-producing antibacterial proteins was first proposed by Eckersall et al. [48,49] for serum amyloid A. The hypothesis was later confirmed by Smolenski et al. [50]. The aforementioned findings of Addis et al. [33], as corroborated more recently by Katsafadou et al. [51], have confirmed the significant involvement of ewes’ mammary glands’ structural units in the defensive process against mastitis-causing bacteria. These findings differ from the classical theory that invading leucocytes were exclusively the defensive cells of ewes against mastitis.

Chiaradia et al. [36] have used 2-D PAGE coupled with a matrix-assisted laser desorption/ionisation time-of-flight mass spectrometer (MALDI-TOF MS) and/or nano LC ESI-LIT MS/MS analysis (LC: liquid chromatography, ESI: electrospray ionisation, LIT: linear ion trap, MS: mass spectrometry) in samples of milk from cases of spontaneous mastitis caused by *Staphylococcus chromogenes*. By using these methods, the authors have described the differential expression and abundance of various proteins in milk whey that could be possibly exploited for use in the diagnosis of the infection [36]. These authors have also suggested that, in ewes, protein detection might be a better diagnostic method than somatic cell counting in terms of accuracy, especially at the early stage of the disease.

Addis et al. [52], in an experimental study of mastitis caused by *Streptococcus uberis*, identified proteins with increasing abundance after mammary infection. These proteins were involved in the innate defence response processes. Among these proteins, lactotransferrin, cathelicidins, calprotectin subunit S100-A9, complement C3 and haptoglobin were identified [52].

Furthermore, Pisanu et al. [53] reported a detailed proteomics analysis in the milk of ewes after intramammary inoculation with *Streptococcus uberis*. During the analysis, these authors identified 287 proteins that were found with differential abundance in samples of mammary secretion [53]; in fact, this is the largest number of proteins that have ever been identified from cases of ovine mastitis. The identified proteins were mostly related to the immune process and the inflammation pathways in the mammary gland. Some of these proteins were pointed out as potential biomarkers for diagnostic purposes. Moreover, the results have provided new hypotheses regarding the defence role of neutrophil extracellular traps in the mammary gland and associated them with mammary epithelial cells [53].

In a more recent study [51], proteomes in the blood and mammary secretion of ewes with experimental mastitis caused by *Mannheimia haemolytica* were studied. In the findings, it emerged that in blood, 33 proteins were identified with differential abundance after challenge: of these, six showed downregulation, 13 showed new appearance and 14 showed varying abundance. Furthermore, in mammary secretion, 89 proteins were identified with differential abundance after challenge: of these, 18 showed downregulation, 53 showed new appearance, three showed upregulation and 15 showed varying abundance. It is noteworthy that 15 proteins showed status changes in the blood and the mammary secretion (Figure 1) [51]. Differential abundance in mammary secretion from inoculated and uninoculated glands (this being the first study that had examined inoculated and uninoculated healthy mammary gland) revealed that 74 proteins were present only in mammary secretion from the inoculated gland. Among the proteins with differential abundance found in mammary secretion, the majority were involved in cell organisation and biogenesis (17 proteins) or in inflammatory and defence response (13 proteins). Moreover, the same study pointed out that cathelicidin-1 was a predominant protein in milk samples examined as soon as 3 hours post-challenge, independently of the infective agent [51]. Therefore, it was proposed as a non-specific diagnostic tool, because (a) there was high correlation with somatic cell counts in milk of affected ewes, (b) it was detected earlier than increased cell counts and (c) as it is not present in milk of healthy ewes, there would be no need to establish a threshold, hence a ‘positive’/‘negative’ assessment would suffice [1].

The entirety of proteomics findings in the various studies is contributing to the elucidation of the pathogenesis of mastitis, particularly with regard to the defence response of ewes. Ewes with mastitis mount a defence response, controlled by many proteins and over various mechanisms and pathways. These are interdependent at various points [51]. Consequently to the production of cytokines by the defence system of the infected animal, neutrophils enter into the infected mammary gland. Katsafadou et al. [51] have indicated the mobility and functionality of neutrophils within the mammary gland may possibly be regulated by proteins, which are involved in cell communications. As part of the process, the degranulation of neutrophils can lead to release of antimicrobial proteins, among which a significant protein is cathelicidin-1 [51]. As well as this, the neutrophils release proteases within the infected gland, which participate in the lysis of proteins in the mammary secretion and cause damage to the mammary parenchyma [54]. The cathelicidins and the proteins of the S100 have a clearly established antimicrobial role; these molecules are produced mainly by neutrophils, as well as by mammary epithelial cells [52,55].

In general, one may suggest that ewes would continue the synthesis and production of milk, at the same time taking into account the significant requirements for the activity of leucocytes within the mammary gland. Various proteins (e.g., serum albumin) participate in this process. According to the theory of energy partitioning in sheep [56,57], the requirements for reproduction (a significant part of which is lactation) take priority over the requirements for the immunological response of the animals. The factors above make evident that, for allocation of energy resources, the continuation of milk production has a higher priority for ewes than the production of defence cells, despite it being a paradox for a ewe with mastitis.

However, it should also be mentioned that differences were also evident in findings between the various proteomics studies. There are many reasons to account for these differences. It is possible that differences in sheep breeds used in the studies under consideration might have led to varying results, as repeated differences in the susceptibility of the various sheep breeds to mastitis have been described [58,59]; this, when coupled with the confirmed differences in the protein content of ewes’ milk [6,29], would have produced varying results. Moreover, variations in the protocols and technical aspects of the experimental and methodological designs can also contribute to the differences found between the results of the various studies. Finally, the variety of causal agents involved in mastitis [10,13] also contributes to the differences in results.

Identification of biomarkers for diagnosis of mastitis is also the fruit of proteomics research into the infection. Cathelicidins have been repeatedly found as being useful for the early and accurate diagnosis of mastitis. Katsafadou [1] has reported a clear correlation between somatic cell counts and the presence of cathelicidin-1 in milk, as well as a good association between the presence of cathelicidin-1 and ovine subclinical mastitis. Indeed, ELISAs (Enzyme-Linked Immunosorbent Assays) have been developed for detection of the protein in the milk of ewes [60]. The validity of detection of cathelicidins for diagnosis of ovine mastitis has been corroborated by Cubeddu et al. [61], who confirmed the proteomics findings by means of immunochemical techniques. Serum amyloid A was also considered as a potential biomarker for diagnosis of mastitis in ewes [51], with ELISAs also having been developed for its detection in milk of ewes [62].

In the study of ovine mastitis, different workers have employed varying technologies for proteomics analysis. Although use of two-dimensional gel electrophoresis has a reasonable cost, it is also tedious and requires increased human involvement and more preparatory steps. However, it has the unique advantage that it can be applied to study proteoforms (or ‘protein species’), which are different forms of the same proteins produced from the genome with a variety of sequence variations, splice isoforms and many post-translational modifications [63,64]. The proteoforms can be seen on 2-DE gels and can be further studied by various techniques (e.g., western blotting). They indicate protein modifications and post-translations as different spots and can thus be separated according to molecular weight and isoelectric point. In contrast, LC-MS/MS can be worked with a high degree of automation and has the clear advantage that it can provide increased output of data and high reproducibility of findings. Moreover, all steps taken with the relevant equipment are now automated, minimising human involvement and interference and making it more user-friendly. Ideally, the two techniques should be considered to be complementary—one providing separation at protein level and enabling analysis of isoforms, whilst the other providing improved output with less labour, but at a significantly higher cost.

## 4. Conclusions

By using proteomics techniques and methodologies, hundreds of proteins can be studied in a single experiment. That way, one can evaluate the dynamics of cellular response to changes in their micro-environment within tissues. It is thus possible to identify changes in protein expression, interaction or modification that occur as a result of changes in normal states and of pathological conditions occurring within that tissue in the host animal. Generation of proteomics data sets may be used to demonstrate the interdependence of the various processes at a cellular level, which are of importance in normal milk production (i.e., cell growth) or in mastitis (i.e., the cellular response to bacterial invasion). Thus, researchers active in mastitis work can view as one picture, in its entirety, the cellular action and response, rather than examining the individual role of each protein separately. Such an experimental approach enables the discovery of associations between cellular processes, which may be used as precursors to new hypotheses.

Using a proteomics approach in milk, one can achieve time separation and identification–characterisation of proteins at the same. Proteomics analysis can help in identifying proteins in healthy or diseased mammary glands, especially the molecular pathways and the cellular functions involved in the production of milk and in the defence response of the mammary gland of ewes. Milk is a ‘tissue’ of great interest that, particularly for ewes, has not been studied in depth.

The use of proteomics approaches will improve the diagnosis of mastitis by establishing biomarkers, the identification of which will serve in the early and accurate diagnosis of the infection. Thus, milk samples from healthy ewes can be distinguished from samples from ewes with mastitis. By identifying proteins with greater significance in the infection, there is the possibility of identifying biomarkers. Furthermore, the pathophysiology of mastitis can be studied in greater depth; this will include protein–protein interactions at various times of the infection. The elucidation of the immune processes can facilitate development of more effective vaccines against the disease, especially vaccines that would enhance non-specific defences of the udder, thus supporting control of all causal agents of mastitis. Nevertheless, there is still a limit to recover the full proteomics data of milk samples due to the limited annotated sheep genome sequences currently available. As identifications in protein data sets in sheep will be extended, proteomics work in mastitis in ewes will also improve in future.
